# Effects of genetically modified soybean on physiological variables and gut microbiota of Sprague-Dawley rats

**DOI:** 10.1371/journal.pone.0311443

**Published:** 2024-12-12

**Authors:** Elham Ashrafi-Dehkordi, Abdolvahab Alborzi, Gholamreza Pouladfar, Seyed Amin Abbasian, Seyed Mohammad Mazloomi

**Affiliations:** 1 Clinical Microbiology Research Center, Nemazee Hospital, Shiraz University of Medical Sciences, Shiraz, Iran; 2 Nutrition Research Center and Department of Food Hygiene and Quality Control, School of Nutrition and Food Sciences, Shiraz University of Medical Sciences, Shiraz, Iran; Wuhan Polytechnic University, CHINA

## Abstract

Soybean is an important source of food and feed. To keep weeds out of soybean it is often genetically modified. The goal of the current study was to evaluate the effects of a diet containing 70% GM soybean on Sprague-Dawley rats. Two groups of rats were fed GM and non-GM soybeans for a period of 120 days, and their body weight, hematology and serum biochemistry were compared. In addition, the effect of the consumption of GM soybean on identified intestinal microbiota and antibiotic resistance was compared with the effect of the consumption of non-GM soybean. Total bacteria and six types of bacteria shared by humans and rats were detected by q-PCR. The results showed that the consumption of GM soybean did not result in any significant changes in body weight, hematology and serum biochemistry. The results of q-PCR indicated that compared with the consumption of non-GM soybeans, the consumption of GM soybean did not have a comparable effect on the abundance of total bacteria, namely Bifidobacterium group, *Clostridium perfringens* subgroup, *Escherichia coli*, Lactobacillus group, and the Bacteroides–Prevotella group. The results of antibiogram showed that the consumption of GM soybean did not change the resistance of *E*.*coli*, although it changed the resistance of *E*. *faecalis* against erythromycin (the GM group was significantly less resistant than non-GM group). Overall, the study indicated that the consumption of GM soybean did not exhibit adverse effects on physiological variables and gut microbiota of rats. However, the obtained antibiogram results indicated that it is necessary to further investigate the antibiotic resistance of the gut microbiota when GM food is consumed.

## Introduction

Genetically modified (GM) crops are widely used in agriculture and food production to improve the agricultural quality and productivity [1]. In 2019, the global area devoted to the cultivation of GM plants reached 190.4 million hectares in 29 countries. 56% of the global biotech crop area is planted in developing countries compared to 44% for industrial countries. Additionally, 42 countries import transgenic crops for feed, food and processing without cultivating transgenic plants [2]. To create resistance to insects or diseases and herbicides, as well as to increase tolerance to salinity and drought stress and extreme temperature stresses, at least one or more genes have been transferred to most commercial transgenic crops [3, 4]. Meanwhile, numerous studies have been conducted to elucidate the composition of GM food and feed, their origin, possible genetic alterations in transgenic crops, as well as to minimize any risks to consumers.

Soybeans are important for both vegetable oil and protein meals and are cultivated worldwide for meals and feed. Meanwhile, the weed threat leading to lower soybean yield has sparked more interest in GM crops [5]. Transgenic herbicide-resistant crops can help increase yield by reducing pressure of weed and minimizing environmental pollution and crop residues. The expression of EPSPS protein from *Agrobacterium tumefaciens* strain CP4 confers tolerance to glyphosate-based herbicides in soybeans [6]. Transgenic soybean consumption is the highest in the world, accounting for 95.9 million hectares of global GM crops, representing 78% of the world’s soybean production [2].

The Herbicide-resistant soybeans containing glyphosate-resistant genes have been cultivated for more than 20 years. Genetically modified organisms (GMOs) in the agri-food system remain a topic of controversy and debate. The safety of GM food has always been a subject of contention, debated by scientists, farmers and consumers [7]. Safety concerns regarding GM food and feed are significant and crucial for their acceptance in the market [8]. Altering the anti-nutritional factors, macro- or micronutrients, and the potential horizontal gene transfer of exogenous transgenes from GM products to human intestinal microbiome or animal intestinal microbiome may result from the random insertion of genes into the plant genome and disruption of gene expression endogenously [9]. The horizontal gene transfer (HGT) from GM crops into gut bacteria is feasible [10]. However, there are concerns about HGT from GM plants to microbes inhabiting the gastrointestinal (GI) tract. So far there have been no reports illustrating the transfer of exogenous transgenes from GM crops to gut microorganisms of animals [11, 12]. Netherwood et al. reported that a small portion of the *epsps* transgene in GM soybean survived passage through the stomach and the small bowel of all the ileostomists, with three out of seven ileostomists the *EPSPS* having transgene within their gut bacteria [13]. The GI tract is the first part of the body that comes into contact with the environment, antigen of food and bacteria, making it the largest immune site in human playing an important role in the metabolic reactions, nutrient absorption and regulation of immune responses [14, 15]. Before GM products enter the market, they must undergo a thorough safety assessment, including possible unexpected effects [16, 17]. Animal experiments provide important and valuable information for the safety of humans and animals eating GM crops. However, many animal studies have been performed to evaluate GM safety, but researchers have evaluated the GM soya safety more based on nutrition, composition, toxicology and allergenicity analyses than based on the principle of equivalence of substances [18–21]. Hence, they have rarely paid attention to intestinal microbiota. There is a link between the function of intestinal microbiota and the increase of metabolic activity, autoimmune diseases and inflammatory diseases. The Intestinal microbiota help control appetite, energy balance, digestion of dietary compounds, supply of micronutrients, and transformation of xenobiotics, survival, proliferation, and prevention of metabolic disorders (e.g. diabetes, obesity), cancers, and cardiovascular diseases [22, 23]. Previous studies have mostly utilized processed materials (such as oil, dehulled/defatted toasted and flour) as the meal, ignoring the fact that food nutritional profile changes under the influence of chemical and physical processes, which have a great impact on the results of animal feeding.

Addressing this research gap instead of pressed soybean meal or flour this study utilizes unprocessed soybeans for direct evaluation of the GM soybean safety. In addition, this study evaluated the health risks related to the long term-feeding of GM soybean diet on Sprague-Dawley (SD)-rats based on examined clinical pathology (body weight, hematology, serum biochemistry, paying more attention to the effects of GM soybean on the rat intestinal microbiota (the quantification and resistance to antibiotics of the bacteria of SD-rats stool).

## Materials and methods

### Ethical statement

Animal care and all experiments procedures were ethically performed following standard protocols approved by the Ethics Committee of Shiraz University of Medical Sciences (IR.SUMS.REC.1399.1342). All experiments and methods were conducted in accordance with the relevant guidelines and regulations. For the anesthesia of animals, a solution of ketamine: xylazine mix [24] was used, based on AVMA Guidelines for the Euthanasia of Animals (AVMA, edition 2020). We declare that all authors have read the checklist of ARRIVE guidelines and complied with its instructions.

### Animal care and management

Eight-week-old SD-rats were obtained from animal laboratory of Center of Comparative and Experimental Medicine of Shiraz University of Medical Science (Shiraz, Iran). Each rat was individually housed in a cage in a facility with controlled air-conditioning, 12-h light/12-h dark cycles, a temperature of 22±2°C, and a relative humidity of (60±5%). The animals had ad libitum access to water and feed. During a two-week adaptation period, all rats were fed a rodent-based diet.

### Experimental design and diet

After adaptation, 24 rats were randomly divided into two groups. The animal feed included the most important GM plant material on the market (RR soybean meal) for the treatment group, and unmodified commercial soybean meal for the control group. Non-GM and GM soybean meals underwent testing for the presence of GM soybean using specific primers for the *EPSPS* (size of 169 bp) and 35S promoter (size of 195 bp) regions (S1 Fig). This genetic control element is found in approximately 95% of the currently commercialized GM crops [25]. The 2 groups were fed diets with 70% (wt/wt) GM soybean or (non-GM-Soybean) traditional soybean respectively. Body weight was measured monthly during the exposure period (S1 Table).

### Hematology and blood biochemistry analysis

Hematology and blood biochemistry were conducted on all surviving rats before the start of the experiment (time 0) and 120th day (the conclusion of the experiment). Hematologic parameters examined included red blood cell count (RBC), hemoglobin (HGB), white blood cell count (WBC), mean corpuscular volume (MCV), hematocrit (HCT), mean corpuscular hemoglobin (MCH), mean corpuscular hemoglobin concentration (MCHC), platelet count (PLT), mean platelet volume (MPV), Lymphocytes (LYM), red cell distribution width (RDW), and platelet distribution width (PDW). These measurements were taken using Blood Cell Counter (NIHON KOHDEN celltac alpha 6500, Japan). Whole blood was collected. Sera was isolated using centrifugation (1500g for 5 min). The sera were analyzed based on a routine clinical chemistry platform (Biotecnica BT1500 Chemistry Analyzer, Italy) in compliance with current standards for quality requirements. Biochemical parameters examined included albumin (Alb), alanine aminotransferase (ALT), aspartate aminotransferase (AST), alkaline phosphatase (ALP), total protein (TP), blood urea nitrogen (BUN), glucose (Glu), calcium (Ca), phosphorus (Ph), creatinine (Cr) with (Autolaser-BT-1500, Italy). Sera samples was also analyzed for the clinical indexes associated with the lipid metabolism, including triglyceride (TG), cholesterol (Chol), total antioxidant capacity (TAC), malondialdehyde (MDA).

### Stool sampling and DNA extraction

Rats stool sampling were performed at two time points (time 0 and 120th day) and were stored in 2-mL sterile tubes at -80c until needed. All the stool samples were obtained directly from the rats’ anuses in the morning. The total DNA of stool samples was extracted using kit (SinaPure ^TM^ DNA, IRAN). A 150 mg stool was washed with 1 mL of TE buffer (10 mM Tris–HCl, 1 mM sodium EDTA, pH 8.0), then was vigorously vortexed for 30 sec. The sample at 10,000×g for 2 min was centrifuged, after that the pellet was suspended in 1 mL of TE buffer, and the washing process was repeated as described above. The sediment of each sample was diluted in 400 μL lysis buffer. We continued the extraction according to the manufacturer’s instructions. Six reference bacterial strains were used for DNA extraction with kit (SinaPure ^TM^ DNA, IRAN). *Bacteroides ovatus* (ATCC 8483), *Lactobacillus casei* (ATCC 393), *Bifidobacterium adolescentis* (ATCC 15703), *Escherichia coli* (ATCC 68233), *Enterococcus faecalis* (ATCC 19433) and *Clostridium perfringens* (ATCC 13124). The extracted DNA quality was assessed with UV spectrophotometry 260 nm to 280 nm wavelengths (ScanDrop2, analytic jena, Germany). The results of an OD260/280 of 1.8 to 1.95 showed that the extracted DNA from stool was theoretically appropriate for PCR reaction.

### Quantitative-PCR

The quantitative-PCR experiments were conducted using an Applied Biosystems StepOne Real-Time PCR System 48 Well. SYBR Green was used for the quantification of total bacteria and 6 types of bacteria common between humans and rats. For each bacteria type, the mean abundance based on q-PCR at the end of 4rd month was compared with that at time 0. Details of q-PCR can be found in (Table 1). A total of 20 μL contained 10 μL of 2 × SYBR Green Master Mix (Ampliqon, Denmark), 1 μL of each primer (final concentration 0.5 μmol/L), and 10 ng of template DNA. The q-PCR reaction was carried out as follows: an initial denaturation step at 95°C for 5 min followed by 40 cycles at 95°C for 30s, an annealing step at the primer-specific temperature (Table 1) for 30s, extension at 72°C for 35s. Subsequently, a melting curve analysis at 95°C for 15s was performed to assess the specificity of PCR reactions. Standard curves were constructed by 10-fold serial dilutions of DNA extracted from the 6 pure strains of known concentrations using the formula C_t_ = a + bx, where b is the slope value, a is the intercept value and x is the unknown value of log_10_ target genome copies (S2 Fig) [15]. The results were expressed as log_10_ target genome copy number g^−1^stool.

**Table 1:** 16S rDNA q-PCR primers used to amplification stool samples.

Target bacteria	Primer 5’- 3’ sequence	Amplicon size (bp)	Annealing temp. (°C)	Reference
Total bacteria	TCCTACGGGAGGCAGCAGT	466	58	[26]
	GGACTACCAGGGTATCTAATCCTGTT			
Bacteroides–Prevotella group	GAAGGTCCCCCACATTG	418	59	[27]
	CAATCGGAGTTCTTCGTG			
Bifidobacterium genus	GGGTGGTAATGCCGGATG	437	60	[27]
	TAAGCGATGGACTTTCACACC			
*Clostridium perfringens*	ATGCAAGTCGAGCGA(G/T)G	120	57	[28]
	TATGCGGTATTAATCT(C/T)CCTTT			
Enterococcus genus	CCCTTATTGTTAGTTGCCATCATT	144	61	[28]
	ACTCGTTGTACTTCCCATTGT			
*E*. *coli* subgroup	GTTAATACCTTTGCTCATTGA	340	61	[29]
	ACCAGGGTATCTAATCCTGTT			
Lactobacillus group	AGCAGTAGGGAATCTTCCA	341	58	[30]
	CACCGCTACACATGGAG			

### Antibiotic resistance

Antibiotic resistance of the *E*. *faecalis* and *E*. *coli* isolated from stool was assessed using the disc-diffusion method. *E*. *coli* and *E*. *faecalis* were inoculated in petri dishes on the Muller-Hinton agar. Mast antibiotic discs (Mast group Ltd, U.K.) of 6 mm in diameter were placed on the surface. These discs were impregnated with (Pen) penicillin (10 units), (Tet) tetracycline (30 μg), (Ery) erythromycin (15 μg), (Kan) kanamycin (30 μg), (Amp) ampicillin (10 μg), (Van) vancomycin (30 μg), (Rif) rifampin (5 μg), (Azi) azithromycin (15μg), (Gen) gentamicin (10 μg). After disc placement the petri dishes inoculated with *E*. *coli* were incubated at 37°C for 24 h under aerobic conditions, while those of *E*. *faecalis* were incubated at 37°C for 48 h under the same conditions. Subsequently, the mean inhibition zone diameters were then compared with the Clinical and Laboratory Standards Institute (CLSI) standards from 2015 to determine the level of antibiotic resistance in the bacterial isolates.

### Statistical analysis

All statistical analyses were performed by the SPSS version 21 statistical computation software package (SPSS Inc., Chicago, IL, USA). The data are expressed as mean ±SD and statistical significance was assigned at the p < 0.05 level. Normality of data distribution was tested with Shapiro-Wilk test. T-test was performed for normal data distribute (Levene’s test for equality of variances was performed) and In cases where the data did not follow a normal distribution, a Mann-Whitney U test was utilized for statistical comparisons.

## Results

### Body weight

**Fig 1:**
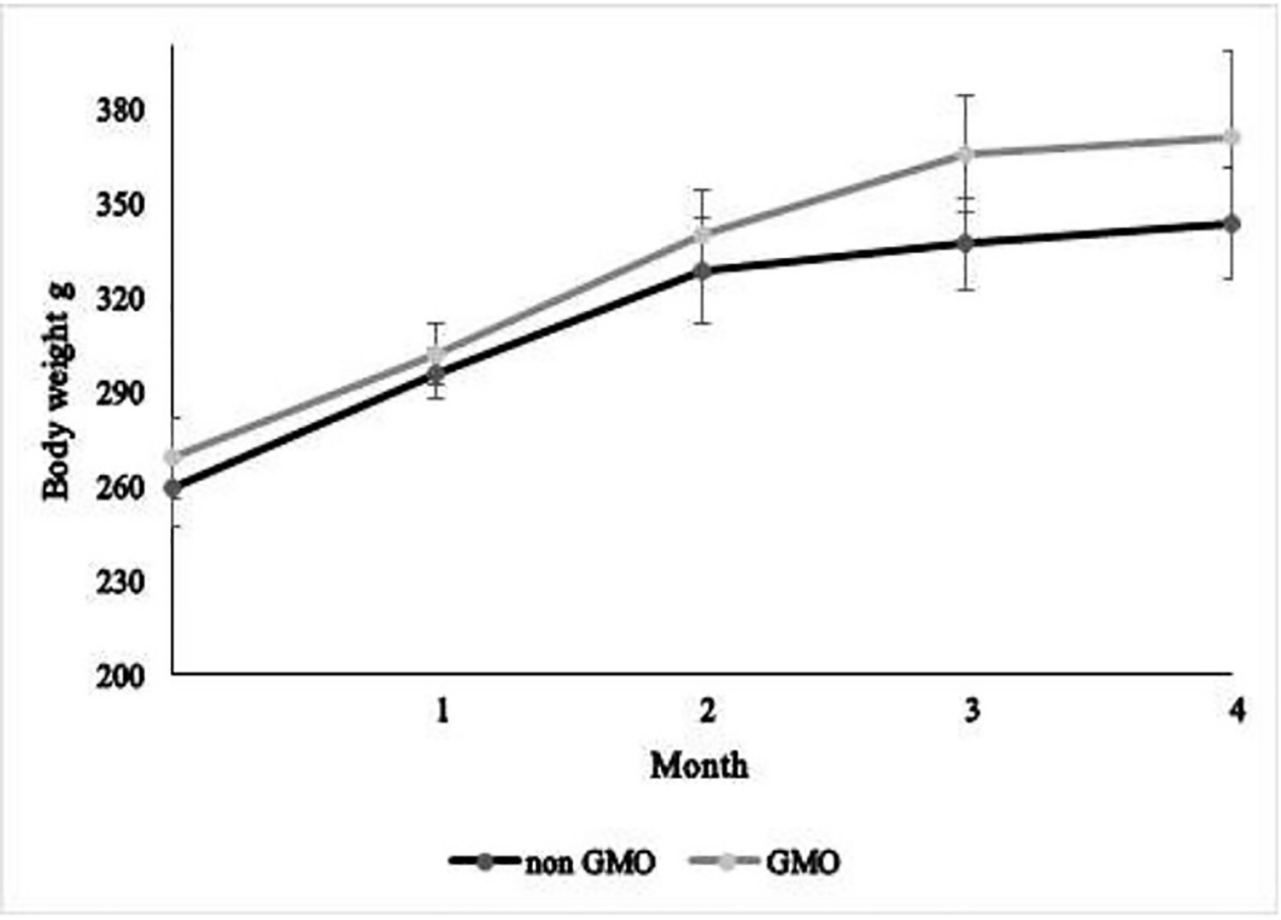
Mean monthly body weight of rats fed with different diets.

There were no significant differences in monthly and final body weights of rats between the GM group and non-GM group (Fig 1).

### Hematology and blood biochemistry

There were no statistically significant differences in hematology variables of rats between the non-GM diet group and GM group (Table 2). As with the hematological variables, there were not any statistically significant difference in serum chemistry variables between the non-GM diet group and the GM group (Table 3).

**Table 2:** Hematology values from two diet groups.

Hematology		Before		After		
Factor	Group	Mean±SD	p-value	Mean±SD	P-value	Mean Difference (*100)
WBC (×10^3^/ μL)	1	9.71±0.0.6	0.22	8.51±1.15a	0.87	1.02
	2	8.83±1.75		8.66±2.23		
RBC (×10^6^/ μL)	1	6.74±0.13	0.77	8.27±0.50a	0.73	-0.21
	2	6.79±0.49		8.11±1.13a		
HGB (g/Dl)	1	13.13±0.44	0.26	14.80±1.04a	0.17	1.04
	2	12.76±0.67		15.47±0.79a		
HCT (%)	1	40.08±1.39	0.73	43.20±3.38	0.29	1.32
	2	40.40±1.82		44.83±2.42a		
MCV (fL)	1	59.45±1.60	0.9	52.17±1.36a	0.24	0.55
	2	59.60±2.50		52.87±1.73a		
MCH (pg)	1	19.48±0.57	0.31	17.88±0.74a	0.39	0.98
	2	18.87±1.49		18.26±0.89		
MCHC (g/dL)	1	30.93±4.58	0.7	34.31±1.56a	0.72	-0.46
	2	31.62±1.83		34.53±0.72a		
PLT (×10^3^/ μL)	1	356.5±20.36	0.22	745.71±143a	0.29	-3.33
	2	378.75±38.62		764.62±62a		
LYM (×10^3^/ μL)	1	5.85±1.34	0.78	5.47±1.26	0.23	1.14
	2	5.63±1.44		6.40±1.83		
RDW (%)	1	10.83±0.60	0.08	14.50±1.59a	0.71	-0.75
	2	11.31±0.33		14.22±1.26a		
MPV (fL)	1	3.95±0.61	0.6	7.11±0.63a	0.18	-0.21
	2	3.81±0.33		6.76±0.32a		
PDW (fL)	1	17.00±0.26	1	8.58±0.98	0.22	-0.49
	2	17.00±0.76		8.08±0.47		

P-value: compared between the non-GM soybean (group 1) and GM soybean (group 2) groups

^a^ Statistically significantly different before and after the experiment in each group (p<0.05)

**Table 3:** Serum chemistry values from two diet groups.

Serum chemistry			Before		After		
Factor		Group	Mean±SD	P-value	Mean±SD	P-value	Mean Difference (100)
Urea (mg/dL)		1	51.85±4.98	0.66	46.71±3.98a	0.08	4.94
		2	50.57±5.68		50.37±3.66		
Cr. (mg/dL)		1	0.51±0.10	0.76	0.50±0.05	0.2	-0.02
		2	0.50±0.05		0.46±0.05		
TG (mg/dL)		1	57.28±9.63	0.003*	68.14±20.07	0.08	-4.80
		2	79.57±12.73		85.62±15.59		
Ca (mg/dL)		1	10.41±0.35	0.08	9.32±0.62a	0.19	-0.36
		2	10.77±0.34		9.32±0.24a		
Chol (mg/dL)		1	54.57±6.21	0.09	55.14±9.88	0.16	-0.03
		2	61.71±8.44		62.25±8.95		
GLU (mg/dL)		1	301.42±36.03	0.62	284.85±51.13	0.72	1
		2	291.57±36.99		276.00±44.68		
Ph. (mg/dL)		1	7.94±1.19	0.83	7.30±3.92	0.72	-1.81
		2	7.82±0.74		5.37±0.26a		
PRO (g/dL)		1	6.27±0.44	0.12	6.21±0.40	0.18	-0.41
		2	6.61±0.32		6.13±0.37		
AST (U/L)		1	124.14±8.15	0.06	76.85±30.32a	0.54	6.23
		2	133.42±8.32		92.37±4.62a		
ALT (IU/L)		1	49.85±10.96	0.58	73.14±13.14a	0.24	-2.08
		2	47.42±2.82		68.62±14.87a		
Alb (g/dL)		1	3.30±0.16	0.01*	2.78±0.18	0.24	-0.11
		2	3.52±0.12		2.90±0.17		
TAC (mM/L)		1	1.32±0.19	0.83	1.27±0.12	0.84	-0.00
		2	1.34±0.16		1.28±0.12		
MDA(μM/L)		1	2.06±0.30	0.66	1.97±0.20	0.87	-0.08
		2	2.13±0.24		1.95±0.30		

P-value: compared between the non-GM (group 1) soybean and GM soybean (group 2) groups

*Statistically significantly different from non- GM soybean (group 1) (P<0.05)

^a^ Statistically significantly different before and after the experiment in each group

### Quantification of bacterial populations in fecal

The Ct had a good linear relationship with the starting amount of DNA (R^2^ > 0.97) (except for *cl*. *perfringens* subgroup that had R^2^>0.92) (S2 Fig). Table 4 shows the results of quantitative-PCR against bacterial 16S rRNA. No statistically significant difference was found between the GM soybean and non-GM soybean groups in the 6 bacterial strains and in the total bacteria counts in stools at the 120-day post-treatment mark. However, by the conclusion of the experiment, the population of lactobacillus significantly decreased in both groups, while the number of Bacteroides increased.

**Table 4:** Real-time PCR quantification of stools samples from rats.

		Before		After		
	Group	Mean±SD	p-value	Mean±SD	p-value	Mean Difference (*100)
Total bacteria	1	10.55±0.42	0.31	10.87±0.40	0.47	-0.24
	2	10.97±0.48		11.05±0.09		
Lactobacillus group	1	10.05±0.39	0.95	5.30±0.84^a^	0.89	0.13
	2	10.02±0.60		5.40±0.82^a^		
*Cl*. *perfringens* subgroup	1	6.31±0.22	0.87	6.20±0.59	0.97	-0.04
	2	6.34±0.23		6.19±0.59		
*E*. *coli* sub group	1	6.43±0.31	0.93	6.58±0.75	0.95	0.06
	2	6.40±0.35		6.61±0.59		
Enterococcus genus	1	5.84±0.92	0.86	6.53±1.73	0.97	0.17
	2	5.72±0.63		6.58±1.87		
Bifdobacterium genus	1	8.18±0.72	0.92	8.22±0.46	0.80	-0.06
	2	8.12±0.81		8.10±0.67		
Bacteroides group	1	8.25±1.43	0.94	8.78±1.85	0.94	-0.03
	2	8.16±1.28		8.66±1.81		

P-value: compared between the non-GM soybean (group 1) and GM soybean (group 2) groups

^a^ Statistically significantly different before and after the experiment in each group

### Antibiotic resistance

*E*. *coli* isolated from the rats stool became more sensitive to all the antibiotics used in two groups (Table 5), while *E*. *faecalis* isolated from the rats stool became more sensitive only to penicillin and erythromycin (Table 5). The effect of GM soybean on the results of susceptibility tests of *E*. *coli* was insignificant. However, in the rats fed with GM soybean susceptible of *E*. *faecalis* to erythromycin, the effect was higher than control group. This difference was statistically significant, indicating a notable variance between the non-GM diet group and the GM group.

**Table 5:** Result of antibiogram of *E*.*coli* and *E*.*faecalis* diameter of the inhibition zone in cm.

***E*. *coli***		Before			After		
Ant.	Group	Mean± Sd (cm)		p-value	Mean ±Sd (cm)	P-value	Mean Difference (*100)
AZM	1	1.90±0.24		0.99	2.96±0.25	0.2	-0.55
	2	1.95±0.17			2.46±0.50		
AP	1	1.93±0.20		0.1	2.16±0.05	0.07	-0.04
	2	1.67±0.23			1.86±0.25		
KAN	1	1.63±0.08		0.24	2.03±0.05	0.09	-0.04
	2	1.57±0.05			1.93±0.05		
TET	1	2.05±0.22		0.15	2.43±0.11	0.25	-0.00
	2	1.82±0.20			2.2±0.26		
GM	1	1.96±0.08		0.69	2.23±0.15	0.10	-0.17
	2	1.97±0.12			2.06±0.05		
***E*.*faecalis***							
PEN	1	1.66±0.32	0.41		1.82±0.13	0.45	-0.05
	2	1.82±0.20			1.92±0.20		
TET	1	2.14±0.20	0.76		2.14±0.15	0.31	-0.06
	2	2.17±0.09			2.11±0.03		
ERY	1	1.98±0.21	0.38		1.88±0.35	0.03*	0.32
	2	2.15±0.33			2.37±0.13		
AMP	1	2.00±0.07	0.37		2.08±0.08	0.68	-0.13
	2	2.20±0.38			2.14±0.32		
VAN	1	1.76±0.11	0.90		1.90±0.14	0.11	-0.14
	2	1.77±0.20			1.77±0.04		
RIF	1	2.02±0.71	0.67		1.43±0.057	0.10	1.25
	2	1.56±0.05			1.64±0.38		

P-value: compared between the non-GM soybean (group 1) and GM soybean (group 2) groups

## Discussion

In the current study, it was found that GM soybean did not exert a significant effect on the monthly and final body weight of the animals. Similar results were obtained by other researchers, for example, Delaney et al. reported that there were no significant difference in food consumption/efficiency and body weight between in rats consuming GM and non-GM meals [19]. Kosieradzka et al. reported observed insignificant differences between the relative weights of some internal organs of rats on a non-GM diet and those fed an additional 10% GM dried potato [31]. In our hematology index results, no significant differences were observed in blood parameters between the non-GM group and the GM experimental group of rats. Several GM crops tested in rat feeding studies have shown similar results, not exhibiting any adverse effects [18, 32–35]. A series of studies showed that dietary GM soybean did not affect the hematological parameters of pigs, calves, broilers, and laying hens [36]. A recent long-term oral toxicity study to assess the safety of transgenic rice in the highly inbred Wuzhishan pigs found no significant difference in the growth, reproduction, haematological parameters or organ morphology between the GM and non-GM groups [37]. Xiang et al. found no significant differences between two transgenic soybean groups and the parental JACK group in survival, growth performance, feed efficiency, body index and enzyme activities after eight weeks of feeding in juvenile channel catfish [38].

No significant difference was observed between the non-GM and the GM experimental group in terms of fecal bacterial quantity indicator. Nejabat et al reviewed 16 scientific publications, in which ten studies (62.5%) used 50% or more GM diet in the treatment group, with the duration of the treatment varying from 7 to 420 days. Nine studies were conducted on rats, four on pigs and saw, two on broilers, and one on mice. In all, eight studies fed their animals genetically modified rice, maize in three cases, corn, apple, soybean, and canola in one case [39]. The review revealed that there was no significant difference in the gut microbiota between the control group and the group on the transgenic diet [39]. Mesnage et al. showed that the consumption of the GM maize varieties (NK603 and MON810) had no effect on the status of the faecal microbiota even up to 33% of the total diet compared to non-GM lines [40]. Xu et al. reported that a diet containing 70% GM rice reduced the abundance of Lactobacillus group. But diets containing 30% and 50% GM rice did not alter the abundance of bacteria [41]. Feeding pigs with transgenic corn (MON810) for 110 days had no effect on total anaerobes and the abundance of *Lactobacillus*, *Enterobacteriaceae*, thought it had an effect on the abundance of the genus *Holdemania* in the caecum [42]. In a separate experiment, Schroder et al. found that feeding rats GM rice resulted in a higher count of coliforms in the ileum, while the count of *bifidobacteria* in the duodenum was lower compared to the control group [43]. Compared with non-transgenic parent wild-type rice, the transgenic variant exhibited no observable impact on the levels of fecal coliform, *lactobacillus* or total anaerobes. Buzoianu et al. demonstrated GM Bt maize had no adverse effects on the intestinal microbiota of pigs and their offspring, though it exerted effects on *Enterobacteriaceae* counts and total anaerobe, as well as in the abundance of *Proteobacteria* [42]. These findings imply that the impact of transgenic feeds on gut microbiota may vary based on the duration of the experiment and the quantity and type of transgenic feed used.

Our findings showed that the number of *lactobacillus* in both groups was significantly lower, while the number of *Bacteroides* group and *Enterococcus* genus higher. Recently, several studies have shown that the population and composition of the gut microbiota may be altered by the consumption of soy foods. Recent research has indicated that soy food consumption can influence the gut microbiota’s population and composition. Findings suggest that soy milk consumption specifically alters the gut’s bacterial makeup, resulting in a heightened presence of the *Bacteroidetes* and *Proteobacteria* groups, alongside a decrease in the relative abundance of *bifidobacteria* and *Firmicutes* [44]. In another study, rats fed chicken protein had higher levels of beneficial genus *Lactobacillus*, while those fed soy protein group had the lowest *Lactobacillus* content [45]. Microflora in the intestine tract is essential for immune, protective, and metabolic functions, with an overall massive impact on the host nutrition and health status.

Considering that Given that mobile elements such as modified DNA can be transferred laterally to other receptors, including eukaryotes, prokaryotes, and even human [46], this study investigated the effect of transgenic soy consumption on the antibiotic resistance of intestinal bacteria, especially on the sensitivity of *E*. *coli* and *E*. *faecalis* bacteria to different antibiotics. The results indicated that *E*. *coli* isolated from rat stool became more sensitive to all antibiotics used in both groups, with no significant effect of GM soybean on the susceptibility tests. Moreover, *E*. *faecalis* isolated from rat stool showed increased sensitivity only to penicillin and erythromycin, with a statistically significant difference between the non-GM diet group and the GM group in terms of erythromycin susceptibility (the resistance was slightly lower at the end of the experiment). Czerwiński et al. reported that the consumption of transgenic corn did not change the resistance of *E*.*coli* and *Clostridium* against antibiotics. However, compared with conventional feeds, GM soybean slightly increased resistance of *clostridium* from caecum against kanamycin and erythromycin and *clostridium* from the ileum against kanamycin [47]. They reported the effect of transgenic feeds on the outcome of susceptibility tests was insignificant. There has been a limited number of studies conducted on susceptibility testing, indicating a significant gap in research. Comprehensive investigations are required to draw definitive conclusions regarding the impact of genetically modified (GM) feed on bacterial resistance to antibiotics.

One of the limitations of this study was the use of one gender of rats. It is suggested that this research be investigated for the female gender as well.

## Conclusions

In conclusion, the consumption of transgenic soy had no significant effect on body weight, serological and biochemical parameters, and microflora of the gastrointestinal tract in rats. However, the consumption of transgenic soybean changed the resistance of E. faecalis against erythromycin. The findings highlights the importance of understanding the potential impact of transgenic soy on antibiotic resistance. Further research is needed to elucidate the mechanisms underlying these interactions and assess the long-term consequences for human health. To ensure the safety and efficacy of these products for consumers, it is necessary to continue investigating the effects of GM foods on the antibiotic resistance of the gut microbiota.

## Supporting information

S1 Fig Representative agarose gel electrophoresis of PCR products. Lanes: N, negative control, non-GMO; P, positive control, GMO and M, 100 bp DNA ladder.
(TIF)

S2 Fig Standard curves for q-PCR method. (TIF)

S1 Table Composition of diets. (DOCX)

## Abbreviation

EPSPS: Enolpyruvate shikimate-3-phosphate synthase
GM: Genetically modified
GMOs: genetically modified organisms
SD: Sprague–Dawley

## References

[1] Ashrafi-Dehkordi E, Alemzadeh A, Tanaka N, Razi H. Effects of vacuum infiltration, Agrobacterium cell density and acetosyringone concentration on Agrobacterium-mediated transformation of bread wheat. JCF. 2021; 16:59–69. 10.1007/s00003-020-01312-y

[2] ISAAA. (2018). Available from: https://www.isaaa.org/

[3] Tsatsakis AM, Nawaz MA, Tutelyan VA, Golokhvast KS, Kalantzi OI, Chung DH, et al. Impact on environment, ecosystem, diversity and health from culturing and using GMOs as feed and food. Food Chemic Toxicol. 2017 Sep 1;107:108–21. doi: 10.1016/j.fct.2017.06.033 28645870

[4] Dehkordi EA, Alemzadeh AB, Tanaka NO. Agrobacterium-MEDIATED Transformation OF Ovary OF BREAD Wheat (Triticum aestivum L.) with A Gene Encoding A TOMATO ERF PROTEIN. *Plant Cell Biotechnol*. *Mol*. *Biol*. 1st–2nd. 2018 Mar 17;19:24–33.

[5] Ferrero R, Lima M, Davis AS, Gonzalez-Andujar JL. Weed diversity affects soybean and maize yield in a long term experiment in Michigan, USA. Front Plant Sci. 2017 Feb 24;8:246616. 10.3389/fpls.2017.00236 PMC5323402 28286509

[6] Ashrafi-Dehkordi E, Mazloomi SM, Hemmati F. A comparison of DNA extraction methods and PCR-based detection of GMO in textured soy protein. JCF. 2021 Mar;16:51–7. 10.1007/s00003-020-01300-2

[7] Xu R, Wu Y, Luan J. Consumer-perceived risks of genetically modified food in China. Appetite. 2020 Apr 1;147:104520. doi: 10.1016/j.appet.2019.104520 31751633

[8] Shi Z, Zou S, Lu C, Wu B, Huang K, Zhao C, et al. Evaluation of the effects of feeding glyphosate-tolerant soybeans (CP4 EPSPS) on the testis of male Sprague-Dawley rats. GM Crops Food. 2019 Jul 3;10(3):181–90. doi: 10.1080/21645698.2019.1649565 31366287 PMC6748360

[9] Yuan Y, Xu W, He X, Liu H, Cao S, Qi X, et al. Effects of genetically modified T2A-1 rice on the GI health of rats after 90-day supplement. Sci Rep. 2013 Jun 11;3(1):1962. doi: 10.1038/srep01962 23752350 PMC3678139

[10] Nawaz MA, Mesnage R, Tsatsakis AM, Golokhvast KS, Yang SH, Antoniou MN, et al. Addressing concerns over the fate of DNA derived from genetically modified food in the human body: A review. Food Chemic Toxicol. 2019 Feb 1;124:423–30. doi: 10.1016/j.fct.2018.12.030 30580028

[11] Uzogara SG. The impact of genetic modification of human foods in the 21st century: A review. Biotechnol adv. 2000 May 1;18(3):179–206. doi: 10.1016/s0734-9750(00)00033-1 14538107

[12] Einspanier R, Lutz B, Rief S, Berezina O, Zverlov V, Schwarz W, et al. Tracing residual recombinant feed molecules during digestion and rumen bacterial diversity in cattle fed transgene maize. Eur Food Res Technol. 2004 Feb;218:269–73. 10.1007/s00217-003-0842-9

[13] Netherwood T, Martín-Orúe SM, O’Donnell AG, Gockling S, Graham J, Mathers JC, et al. Assessing the survival of transgenic plant DNA in the human gastrointestinal tract. Nature biotechnol. 2004 Feb 1;22(2):204–9. doi: 10.1038/nbt934 14730317

[14] Laparra JM, Sanz Y. Interactions of gut microbiota with functional food components and nutraceuticals. Pharmacol Res. 2010 Mar 1;61(3):219–25. doi: 10.1016/j.phrs.2009.11.001 19914380

[15] Yuan Y, Xu W, He X, Liu H, Cao S, Qi X, et al. Effects of genetically modified T2A-1 rice on the GI health of rats after 90-day supplement. Sci Rep. 2013 Jun 11;3(1):1962. doi: 10.1038/srep01962 23752350 PMC3678139

[16] Myskja BK, Myhr AI. Non-safety assessments of genome-edited organisms: should they be included in regulation?. Sci Eng Ethics. 2020 Oct;26(5):2601–27. doi: 10.1007/s11948-020-00222-4 32424723 PMC7550366

[17] Kleter GA, Kok EJ. Safety assessment of biotechnology used in animal production, including genetically modified (GM) feed and GM animals-a review. Ani Sci Paps Rep. 2010;28(2):105–14.

[18] Appenzeller LM, Munley SM, Hoban D, Sykes GP, Malley LA, Delaney B. Subchronic feeding study of herbicide–tolerant soybean DP-356Ø43-5 in Sprague–Dawley rats. Food chem Toxicol. 2008 Jun 1;46(6):2201–13. 10.1016/j.fct.2008.02.017. 18403083

[19] Delaney B, Appenzeller LM, Munley SM, Hoban D, Sykes GP, Malley LA, et al. Subchronic feeding study of high oleic acid soybeans (event DP-3Ø5423-1) in Sprague–Dawley rats. Food chem Toxicol. 2008 Dec 1;46(12):3808–17. 10.1016/j.fct.2008.10.003 18952136

[20] Hammond BG, Vicini JL, Hartnell GF, Naylor MW, Knight CD, Robinson EH, et al. The feeding value of soybeans fed to rats, chickens, catfish and dairy cattle is not altered by genetic incorporation of glyphosate tolerance. J Nut. 1996 Mar 1;126(3):717–27. 10.1093/jn/126.3.717 8598557

[21] Qian ZY, Zhang SJ, Zhang L, Zhang J, Liu YH, Zhou QH, et al. Subchronic toxicity study in rats evaluating genetically modified DAS-81419-2 soybean. RTP. 2018 Jul 1;96:48–56. doi: 10.1016/j.yrtph.2018.04.019 29715492

[22] Sommer F, Bäckhed F. The gut microbiota—masters of host development and physiology. Nature Rev Microbiol. 2013 Apr;11(4):227–38. doi: 10.1038/nrmicro2974 23435359

[23] Salonen A, de Vos WM. Impact of diet on human intestinal microbiota and health. Ann Rev Food Sci Technol. 2014 Feb 28;5:239–62. doi: 10.1146/annurev-food-030212-182554 24387608

[24] Underwood W, Anthony R. AVMA guidelines for the euthanasia of animals: 2020 edition. Retrieved on March. 2020 Mar;2013(30):2020–1.

[25] Forte VT, Di Pinto A, Martino C, Tantillo GM, Grasso G, Schena FP. A general multiplex-PCR assay for the general detection of genetically modified soya and maize. Food Control. 2005 Jul 1;16(6):535–9. https://doi.org/10.3389%2Ffmicb.2015.00757

[26] Nadkarni MA, Martin FE, Jacques NA, Hunter N. Determination of bacterial load by real-time PCR using a broad-range (universal) probe and primers set. Microbiol. 2002 Jan;148(1):257–66. doi: 10.1099/00221287-148-1-257 11782518

[27] Bartosch S, Fite A, Macfarlane GT, McMurdo ME. Characterization of bacterial communities in feces from healthy elderly volunteers and hospitalized elderly patients by using real-time PCR and effects of antibiotic treatment on the fecal microbiota. Appl Anviron Microbiol. 2004 Jun;70(6):3575–81. doi: 10.1128/AEM.70.6.3575-3581.2004 15184159 PMC427772

[28] Rinttilä T, Kassinen A, Malinen E, Krogius L, Palva A. Development of an extensive set of 16S rDNA‐targeted primers for quantification of pathogenic and indigenous bacteria in faecal samples by real‐time PCR. J Appli Microbiol. 2004 Dec 1;97(6):1166–77. doi: 10.1111/j.1365-2672.2004.02409.x 15546407

[29] Malinen E, Kassinen A, Rinttila T, Palva A. Comparison of real-time PCR with SYBR Green I or 5′-nuclease assaysand dot-blot hybridization with rDNA-targeted oligonucleotide probes in quantificationof selected faecal bacteria. Microbio. 2003 Jan;149(1):269–77. doi: 10.1099/mic.0.25975-0 12576600

[30] Walter J, Hertel C, Tannock GW, Lis CM, Munro K, Hammes WP. Detection of Lactobacillus, Pediococcus, Leuconostoc, and Weissella species in human feces by using group-specific PCR primers and denaturing gradient gel electrophoresis. Appl Environmen Microbiol. 2001 Jun 1;67(6):2578–85. 10.1128/AEM.67.6.2578-2585.2001 PMC92910 11375166

[31] Kosieradzka I, Sawosz E, Szopa J, Bielecki W. Potato genetically modified by 14-3-3 protein repression in growing rat diets. Part II: Health status of experimental animals. Polish J food Nut Sci. 2008;58(3).

[32] Malley LA, Everds NE, Reynolds J, Mann PC, Lamb I, Rood T, et al. Subchronic feeding study of DAS-59122-7 maize grain in Sprague-Dawley rats. Food Chemic Toxicol. 2007 Jul 1;45(7):1277–92. doi: 10.1016/j.fct.2007.01.013 17329002

[33] Teshima R, Okunuki H. A, Sakushima J.I, Goda Y, Onodera H, Sawada J.I, et al. “Effect of GM and nonGM soybeans on the immune system of BN rats and B10A mice. J Food Hyg Soc Jpn, 2000; 41:188–193.

[34] Hammond B, Dudek R, Lemen J, Nemeth M. Results of a 13 week safety assurance study with rats fed grain from glyphosate tolerant corn. Food Che Tox. 2004 Jun 1;42(6):1003–14. doi: 10.1016/j.fct.2004.02.013 15110110

[35] Hammond B, Lemen J, Dudek R, Ward D, Jiang C, Nemeth M, et al. Results of a 90-day safety assurance study with rats fed grain from corn rootworm-protected corn. Food Che Tox. 2006 Feb 1;44(2):147–60. doi: 10.1016/j.fct.2005.06.008 16084637

[36] Bednarek D, Dudek K, Kwiatek K, Świątkiewicz M, Świątkiewicz S, Strzetelski J. Effect of a diet composed of genetically modified feed components on the selected immune parameters in pigs, cattle, and poultry. J Vet Res. 2013 Jan 1;57(2):209–17. 10.2478/bvip-2013-0038

[37] Liu Q, Wu S, Li M, Yang W, Wang Y, Wu Y, et al. Effects of long-term feeding with genetically modified Bt rice on the growth and reproductive performance in highly inbred Wuzhishan pigs. Food control. 2018 Aug 1;90: 382–91. 10.1016/j.foodcont.2018.03.017

[38] Xiang D, Luo M, Jiang F, Wen Z, Chen X, Wang X, et al. Safety assessment of subchronic feeding of insect-resistant and herbicide-resistant transgenic soybeans to juvenile channel catfish (Ictalurus punctatus). Sci Rep. 2023 Apr 3;13(1):5445. doi: 10.1038/s41598-023-31072-2 37012256 PMC10070625

[39] Nejabat M, Heydari M, Keshani P, Joulaei H, Nazari N. Effects of Genetically Modified Food on Gut Microbiota in Animal Models: A Systematic Review. JHSSS. 2024 Jan 1;12(1):1–1.

[40] Mesnage R, Le Roy CI, Biserni M, Salles B, Antoniou MN. Relationship between faecal microbiota and plasma metabolome in rats fed NK603 and MON810 GM maize from the GMO90+ study. Food Che Tox. 2019 Sep 1;131:110547. doi: 10.1016/j.fct.2019.05.055 31170423

[41] Xu W, Li L, Lu J, Luo Y, Shang Y, Huang K. Analysis of Caecal Microbiota in Rats Fed with Genetically Modified Rice by Real‐Time Quantitative PCR. J food Sci. 2011 Jan 76(1):M88–93. doi: 10.1111/j.1750-3841.2010.01967.x 21535699

[42] Buzoianu SG, Walsh MC, Rea MC, O’Sullivan O, Crispie F, Cotter PD, et al. The effect of feeding Bt MON810 maize to pigs for 110 days on intestinal microbiota. PLoS One. 2012 May 4;7(5):e33668. doi: 10.1371/journal.pone.0033668 22574106 PMC3344822

[43] Schrøder M, Poulsen M, Wilcks A, Kroghsbo S, Miller A, Frenzel T, et al. A 90-day safety study of genetically modified rice expressing Cry1Ab protein (Bacillus thuringiensis toxin) in Wistar rats. Food Chem Toxicol. 2007 Mar 1;45(3):339–49. doi: 10.1016/j.fct.2006.09.001 17050059

[44] Fernandez-Raudales D, Hoeflinger JL, Bringe NA, Cox SB, Dowd SE, Miller MJ, et al. Consumption of different soymilk formulations differentially affects the gut microbiomes of overweight and obese men. Gut Mic. 2012 Nov 16;3(6):490–500. doi: 10.4161/gmic.21578 22895080 PMC3495786

[45] Yang X, Liang Q, Chen Y, Wang B. Alteration of methanogenic archaeon by ethanol contribute to the enhancement of biogenic methane production of lignite. Front microbiol. 2019 Oct 10;10:2323. doi: 10.3389/fmicb.2019.02323 31649654 PMC6796574

[46] Lerner A, Benzvi C, Vojdani A. The Potential Harmful Effects of Genetically Engineered Microorganisms (GEMs) on the Intestinal Microbiome and Public Health. Microorganisms. 2024 Jan 23;12(2):238. doi: 10.3390/microorganisms12020238 38399642 PMC10892181

[47] Czerwiński J, Śliżewska K, Korwin-Kossakowska A, Bachanek I, Smulikowska S. Effects of genetically modified maize and soybean meal on the diversity and activity of gut microbiota in broiler chicken. Anim Sci Pap Rep. 2017 Jan 1;35(3):279–99.

