# Supplementary figures and images for: Effects of genetically modified soybean on physiological variables and gut microbiota of Sprague-Dawley rats
Source: PLoS One. 2024 Dec 12;19(12):e0311443. doi: 10.1371/journal.pone.0311443 (PMC11637389; doi:10.1371/journal.pone.0311443)

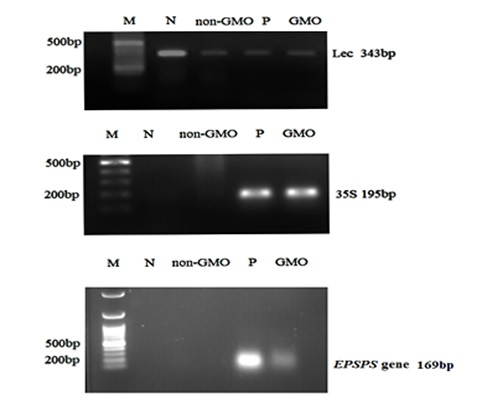

Supplement: S1 Fig — Lanes: N, negative control, non-GMO; P, positive control, GMO and M, 100 bp DNA ladder. (TIF) [file pone.0311443.s001.tif]

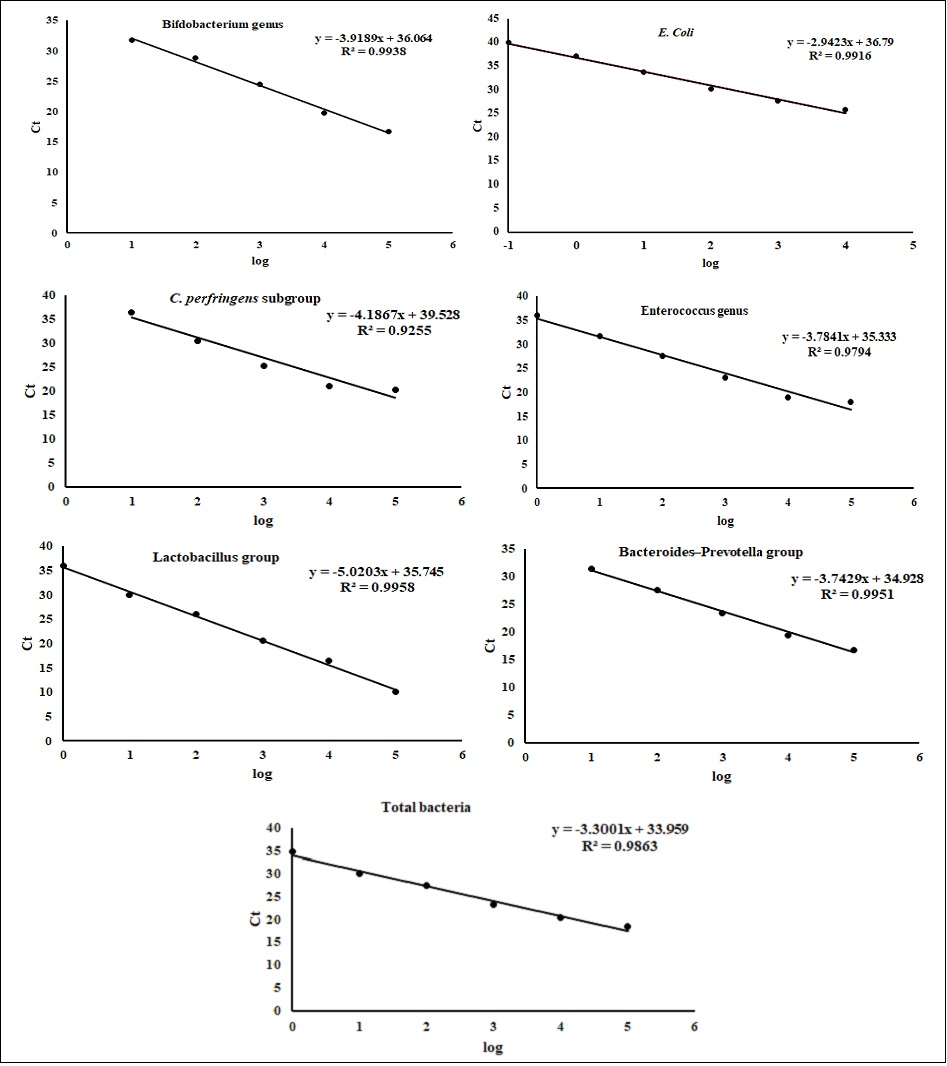

Supplement: S2 Fig — (TIF) [file pone.0311443.s002.tif]
