# Supplementary material for: Effects of genetically modified soybean on physiological variables and gut microbiota of Sprague-Dawley rats
Source: PLoS One. 2024 Dec 12;19(12):e0311443. doi: 10.1371/journal.pone.0311443 (PMC11637389; doi:10.1371/journal.pone.0311443)
Supplement: S1 Table — (DOCX) [file pone.0311443.s003.docx]

| Ingredient | GMO diet | Non-GMO diet |
| --- | --- | --- |
|  | g/kg diet | |
| GM-Soybean | 700 | - |
| Non-GM Soybean | - | 700 |
| Sucrose | 278.8 | 278.8 |
| Mineral mix (AIN-93-MX) | 16.64 | 16.64 |
| Vitamin mix (AIN-93-VX) | 0.252 | 0.252 |
| L-Cystine | 1.8 | 1.8 |
| Choline bitartrate (41.1% choline) | 2.5 | 2.5 |
| tert-Butylhydroquinone (TBHQ), | 0.008 | 0.008 |

Table S1. Composition of diets
